# The hidden impact of workforce instability on patient trust

**DOI:** 10.3389/frhs.2026.1751923

**Published:** 2026-02-26

**Authors:** Waseem Jerjes, See Chai Carol Chan, Marcin Klingbajl, Azeem Majeed

**Affiliations:** Department of Primary Care and Public Health, Faculty of Medicine, Imperial College London, London, United Kingdom

**Keywords:** continuity of care, person-centred care, primary care, trust, workforce stability

## Introduction

Workforce instability refers to recurrent disruption in who delivers care, when care is delivered, and whether adequate capacity is available—arising from vacancies, staff turnover, rota gaps, reliance on temporary staff, and short clinician placements. Yet policy too often frames workforce instability as an administrative or financial issue rather than a determinant of the trust that patients have in health services. But for patients who need to use health services frequently—such as those with complex multimorbidity—workforce instability can have major effects on the continuity of care they receive and their trust in their healthcare providers.

This relationship is fundamentally person-centred: person-centred care is built on understanding the person's priorities, context and lived experience, involving them in decisions, and sustaining a therapeutic relationship over time. When the workforce is unstable, patients are less likely to feel known, heard, and involved, and the service becomes harder to navigate as a coherent “care relationship” rather than a series of transactions. In this way, workforce instability is not only an operational problem; it undermines the conditions required for person-centred care and thereby weakens trust (1–3).

We use workforce instability to mean recurrent disruption in who delivers care, when care is delivered, and whether adequate capacity is available. For clarity, we distinguish five overlapping dimensions: (1) turnover and short placements (high frequency of clinician change); (2) rota volatility (unpredictable scheduling, cancellations, and last-minute changes); (3) understaffing and capacity gaps (vacancies, sickness absence, and unfilled sessions); (4) reliance on temporary staffing (locums/bank/agency staff with variable integration into teams); and (5) fragmentation across sites and services (staff and patients moving between settings with weak information transfer). These dimensions matter because they disrupt different forms of continuity and may therefore affect patient trust through different mechanisms.

Furthermore, we distinguish trust from trustworthiness. Trust refers to a patient's willingness to accept vulnerability in a clinical relationship or system, including uncertainty about outcomes and dependence on others' actions. Trustworthiness refers to the perceived attributes of the clinician, team, or organisation that invite trust, commonly framed as competence, integrity, and benevolence. Hence, workforce instability can affect trust directly by disrupting relationship-building, and indirectly by weakening the signals of trustworthiness that support person-centred care (for example, continuity, follow-through, clear accountability, and the patient's sense that their values and preferences are carried forward). Throughout, we use trust for the patient's willingness to rely on care, and trustworthiness for the qualities and signals patients evaluate in clinicians, teams, and systems (4–6).

In many high-income systems, concerns about health-worker shortages have grown despite increases in staffing (7). In England, the NHS Long Term Workforce Plan sets out growth and retention plans, but short-term instability persists in terms of services coping with demand spikes and recruitment challenges (8). Overall headcounts have risen but pressure remains unevenly distributed across professions and places (9). As of 31 March 2025, the medical staff vacancy rate in England was 4.8% (7,679 posts), a reduction from 5.7% a year earlier but still significant for day-to-day service delivery (10).

Regulators continue to report persistent difficulty in securing stable staffing across sectors (11). Such system indicators matter because a patient's experience of care depends not only on access, but also on seeing the same clinicians and on stable clinical teams. In this opinion paper, we discuss why continuity and trust are core person-centred, quality and safety measures and not just administrative measures.

Nationally, reported trust remains high: in the 2025 GP Patient Survey, 92.5% of respondents had confidence and trust in the clinician they spoke with or saw, from more than 700,000 responses (12, 13). But trust with clinicians will be frail where relationships are continually interrupted. Relational continuity—seeing clinicians who know a patient's history—underpins trust and safe decision-making. Yet relational continuity has been declining in many settings (14, 15).

An extensive body of empirical research links continuity to key clinical outcomes. A systematic review found that higher continuity is associated with lower mortality across diverse health systems (16); English studies show that greater continuity is associated with fewer ambulatory care–sensitive admissions among older people (17); and primary care analyses consistently report that higher physician continuity is linked to lower total costs and reduced hospitalisations (18).

Instruments for measuring patient-trust, such as the Primary Care Assessment Survey, make explicit how interpersonal process and organisational characteristics translate to trust values at the level of the clinician (19, 20). In combination, the collections of evidence here suggest instability is not simply an operational nuisance; it is a threat to the signals of trustworthiness created through continuity, reliability and accountability, and may affect the outcomes we already measure (20–22).

The hidden trust consequences of workforce instability deserve the same attention as metrics such as access and efficiency. Hence, we describe the mechanisms through which instability may erode patient trust, and weaken perceived trustworthiness, the operational ways in which patients experience it, and practical system- and service-level approaches that shield patients from its impact without placing blame on staff. The aim, here, is to re-position continuity and trust as core safety and equity outcomes rather than peripheral measures of patient experience.

### Why trust is fragile when the workforce is unstable: mechanisms and evidence

Patient trust is relational and develops cumulatively rather than emerging from a single encounter; it reflects a willingness to rely on clinicians and services under conditions of uncertainty. Syntheses from health policy and psychology show that trust is shaped by perceived trustworthiness, including competence, integrity (for example honesty and fidelity), and benevolence (for example caring), and that it builds across repeated interactions in which expectations are consistently met (23–27). When staffing is unstable, these repeated interactions are disrupted; patients face new clinicians, different styles, and uncertain accountability. Qualitative research in primary care highlights that “secure trust” arises when patients see the same clinician over time, whereas single-episode care forces reliance on a thinner form of “institutional trust” in the medical system (27). These dynamics illustrate why instability is fundamentally a relational issue, with consequences for disclosure, adherence and shared decision-making—core processes through which person-centred care is enacted in routine practice.

In practice, turnover and short placements primarily disrupt relational continuity, rota volatility and understaffing undermine management continuity (follow-through and accountability), while temporary staffing and cross-site fragmentation particularly threaten informational continuity unless records, handovers and team structures are deliberately designed to preserve narrative coherence.

At a system level, workforce instability is shaped by training pipelines, labour markets, contractual models, and funding and regulatory incentives that influence vacancies, turnover and rota gaps. These upstream drivers often manifest locally as uneven staffing across regions and professions, and as short-term responses to demand spikes that prioritise coverage over continuity. When system pressures repeatedly disrupt access and follow-through, they can weaken institutional signals of trustworthiness (for example, predictability and accountability), even where individual clinicians perform well (4, 9, 15).

At an organisational and team level, instability becomes operational: how rotas are built, how temporary staff are inducted, how results are owned, and whether the record preserves narrative memory. These are design choices that determine whether informational and management continuity survive clinician change. Stable micro-teams, clear ownership for follow-up, and reliable handovers can preserve coherent plans and reduce “plan drift”, thereby protecting perceived trustworthiness even when relational continuity is imperfect (6, 14, 19, 28).

At an individual (patient) level, the consequences are experienced as effort, uncertainty and risk: repeated retelling, inconsistent advice, and unclear responsibility for next steps—all of which increase “patient work” and reduce the likelihood that care feels person-centred, coherent and tailored to what matters to the patient. These experiences influence whether patients feel safe to disclose, whether they re-attend, and whether they rely on the service when problems arise. The effect is often greatest for people with complex multimorbidity, frailty, or social vulnerability, for whom continuity reduces cognitive load and supports shared decision-making over time (5, 11, 16, 29).

Continuity offers stability in the midst of workforce turbulence. It comprises relational continuity, informational continuity, and management continuity with each contributing a distinct dimension to quality and safety (20). Tools such as the Usual Provider of Care index translate these concepts into measurable indicators of whether patients receive care from stable clinicians or teams (22, 28, 29). However, rota gaps, turnover and reliance on temporary staff disrupt all three forms: narrative memory fragments and clear ownership of a patient's care is lost. When patients lack a sense of consistent stewardship, they may disclose less, attend less reliably, and follow plans less consistently, which may compromise safety and quality.

Relational continuity supports trust through recognition, accumulated understanding, and the expectation that the clinician (or team) “knows me”. Informational continuity supports trust through preserved narrative memory: the record and team knowledge maintain context, values, prior reasoning, and what has (and has not) worked, so that advice remains coherent across contacts. Management continuity supports trust through follow-through: clear ownership of tasks, consistent plans across settings, and reliable safety-netting and results management. Workforce instability can threaten all three, but not always to the same extent (4, 9, 15, 30).

In urgent or episodic care, informational and management continuity may be the dominant protectors of trust, because patients primarily need coherent decisions and dependable follow-through even if they do not see the same clinician. In long-term conditions, frailty, mental health, and multimorbidity, relational continuity often becomes more central because repeated negotiation, disclosure, and shared decision-making are cumulative. A practical implication is that services can preserve trust under staffing volatility by prioritising the continuity domain most at risk in that context—for example, strengthening narrative memory and ownership mechanisms where relational continuity cannot be guaranteed (5, 10, 19, 31).

Much of the evidence linking continuity, trust, safety and outcomes is observational and therefore demonstrates association rather than causation. Hence, cautious language should be used (for example, “is associated with”, “may contribute to”, and “is consistent with”) when describing consequences of instability. The mechanisms described are plausible and supported by qualitative and quantitative studies, but the magnitude of effect and the impact of specific interventions are likely to vary by setting, baseline continuity, and patient group (6, 15, 23, 32).

A substantial empirical literature demonstrates the connection between continuity and trust. At the interpersonal level, patients who see a regular clinician report significantly higher trust (19, 21, 26, 30). At the system level, continuity is also associated with outcomes that reinforce trust: One study link higher continuity with fewer ambulatory care–sensitive hospitalisations among older people (17), and another show that patients with stronger physician continuity incur lower costs and experience fewer hospitalisations (18). Although observational in nature, the consistency of these findings across different health systems supports the inference that continuity shapes both measurable outcomes and the relational climate that enables trust. Consequently, workforce instability may weaken patient trust by disrupting repeated interactions and by reducing the perceived trustworthiness of care (for example, follow-through, coherence, and clear accountability). Together, these domains operationalise person-centred care in practice: they support being known over time, carrying preferences and context forward, and ensuring agreed plans are reliably enacted.

Continuity is not valuable simply because patients see a familiar face. It reduces information asymmetry, aids clinicians in recognising a patient's usual patterns, helps identify early signs of deterioration, and provides a stable foundation for honest communication when plans change or errors arise. Evidence links strong continuity with core outcomes of effective primary care, including better population health and more equitable service use, indicating that patient trust is also a marker of system performance (25, 31, 32). Where personal continuity cannot be guaranteed, trust can still be supported through clearly defined teams and structured handovers but only if services intentionally design for reliability rather than hoping it emerges despite staffing turnover.

### From rota gaps to ruptured relationships: operational pathways that damage trust

Different dimensions of workforce instability affect patients through several routes. The first is scheduling disruption (a form of rota volatility), which primarily threatens management continuity and often relational continuity when patients cannot rebook with the same clinician or team. Cancelled clinics, last-minute changes, and unfamiliar clinicians reset plans, break the narrative of care, and make accountability difficult to establish. Patients learn not to expect continuity and interactions become transactional rather than cumulative, reducing the sense of person-centred care because priorities, preferences and context are less likely to be recognised and carried forward (33, 34). This is more than an inconvenience: relational, informational and management continuity all depend on stable contact over time, shared narrative memory and consistent plans. These are elements that are undermined when the workforce is fluid (20, 24, 25, 32). The NHS in England continues to report sustained operational pressures and difficulty maintaining stable staffing across services, making such disruptions routine rather than exceptional (8). Even when headline access appears to be maintained, repeated interruptions shift the patient experience from “someone here knows me” to “no one owns my care,” a transition that may erode patient trust over time.

A second pathway is handover friction. When teams are unstable—through turnover, rota gaps or excessive use of temporary workers—information is transferred under the pressure of documentation more than through “warm” relational handover. Without time and planning for handovers, informational continuity is most directly compromised: problem lists become disjointed, the context for earlier decisions is lost, and safety netting is generic, not bespoke. Continuity literature is emphatic about the point that patients test trust, in judging the doctor, not just in technical expertise, but in coherence—whether advice today is consistent with yesterday's plan and the follow-up planned for tomorrow (19, 20, 33, 35, 36). Where coherence is lacking, the patient interprets indifference or incoherence even where the individual doctor is operating at the edge of their ability.

Third, instability places more cognitive and emotional demand upon patients, reflecting failures of informational continuity (patients must rebuild the story) and management continuity (plans feel inconsistent or unfinished). Re-relating complex histories, reconciling disparate advice and coping with fluctuating expectations are exhausting. Qualitative evidence has shown trust is strengthened where patients feel understood and acknowledged (27, 34–36). It is most necessary in people who have multimorbidity or who are most socially vulnerable, where relational continuity is most essential in enabling them to plan care and make sense of trade-offs. The broader primary care literature associates strong, person-centred primary care and better population health and more equitable utilisation; symmetrically, continuity loss through instability risks exacerbating inequities in experience and outcome (25, 31, 36, 37).

As indirect indicators of trust, they are nonetheless downstream manifestations of the very same mechanism: stable relationships produce better anticipation, earlier correction of course and safer safety netting. In the presence of volatile staffing, though, management continuity can weaken—follow-through deteriorates, test results return to unfamiliar clinicians, and named responsibility blurs. Patients also often revert to urgent care which creates problems such as fragmentation of care, increased costs and extra pressures on emergency health services (21, 37, 38).

Policy analyses have cautioned that measured continuity decreases over time in most settings (14, 15, 39); patient reported trust and confidence remain strong in the round, but those bulk statistics can belie local variation where instability is concentrated (12, 13, 38). The practical message is not to censure individuals rotating or filling gaps, but to note that instability is consistently associated with weaker continuity and may contribute to reduced patient trust in the absence of conscious design (40).

### Designing for trust when staffing is volatile: pragmatic solutions

The approaches vary in the strength of supporting evidence. Some (for example, team-based continuity, structured handovers and continuity measurement) are established practices with substantial implementation experience, while others (for example, brief “continuity notes” and real-time trust dashboards) are proposed or emerging approaches that are plausible but require formal evaluation (4, 5, 41, 42).

Workforce instability cannot be avoided but its effects need not always be directly felt by patients. Designing for reliability of story and of relationship is the organising principle, so that trust is maintained—and person-centred care remains possible—even when the individual doctor moves on. It starts at empanelment and at team-based continuity: each patient is anchored in a small team who share information, routine and responsibility. When someone familiar is indisposed, someone else who is recognised covers; ownership is maintained, and patients get to know who “their team” is rather than taking a bet about who is in the rota today.

In primary care, this fits best with evidence that continuity improves outcomes (16–18, 26, 33) and policy commentary arguing services must measure and manage continuity explicitly rather than hoping it will develop anyway (14, 15, 39). In practice, continuity can be tracked using the Usual Provider of Care (UPC) index (proportion of a patient's contacts with their most-seen clinician) and reviewed quarterly at list and sub-cohort level (22, 43). In hospital settings, a similar “named team” approach can make the most of relationships across admissions and clinics, conveying accountability in the face of rota churn, and responding to the concerns of the regulators about persistent staffing pressures (11). Team-based continuity and continuity measurement are already used in many systems and can be implemented using existing operational data, even where the trial evidence base is limited (44, 45). By contrast, specific tools as “innovations to test” (for example, a Trust Early-Warning System and brief continuity notes) are proposed mechanisms for evaluation rather than established interventions (20, 23, 40, 45).

Reliability also depends on effective handovers and a preserved narrative memory. Handovers can be standardised to include a concise narrative, current objectives and likely next steps rather than a simple data transfer as patients experience this as care that “remembers” them. All three forms of continuity (relational, informational and management) can be made explicit and visible in routine practice (20, 40, 41). Displaying these threads in everyday work through shared problem lists, clear summaries of planned management, and “personal continuity” notes about what matters to the patient enables the next clinician to maintain the tone and direction of care. Because trust is grounded in perceived competence, fidelity and care, making reasoning and follow-through transparent helps preserve the interpersonal foundations of trust even when clinicians change (19, 23, 24, 27, 34).

Rostering can be tuned to safeguard continuity without unduly compromising access. Small, but collectively substantial, levers are: sequence clinics so patients are predominantly booked with their Regular Clinician or Team; hold back a proportion of capacity for follow-up by the same team; and avoid patterns that disperse a clinician's list across numerous microsites. Feedback can inform such decisions. Services may use continuity indices (e.g., the Usual Provider of Care and allied measures) at baseline to set realistic objectives and track change (14, 22, 35, 36). Measure patient reported trust and confidence, in established survey instruments at service level (12, 13, 26, 37), and couple them with operational indicators like re-attendance and complaint themes in identifying where instability is undermining the bond. Publishing plain run charts to staff and boards converts continuity and trust from “soft” experience measures back into transparent system performance.

When instability cannot be avoided, the first priority may be to protect high-need groups. Identify cohorts for whom relational continuity provides the greatest marginal benefit such as people living with frailty, complex multimorbidity, palliative care needs or severe mental illness. Then secure team continuity and proactive follow-up for these groups, drawing on evidence that strong primary care improves population health and reduces inequities (25, 38, 42). Temporary staff can be adequately integrated into clinical teams rather than “parachuted in”: they need a concise, standardised introduction to team routines, access to templates that preserve narrative coherence and clear escalation pathways so that responsibility remains visible to patients. Improving staff retention also supports trust by promoting predictable patterns of care, effective supervision and mentorship, and fewer ruptures in clinician-patient relationships (7, 8).

None of these measures blame individuals for system instability. They recognise that trust is built by design—through visible ownership, coherent stories, and reliable teams—so that even in unstable conditions, patients can answer two questions affirmatively: who is responsible for my care, and can I trust them to follow through?.

### From solutions to method: practical innovations to test

In addition to established continuity and handover practices, innovations that make trust and continuity measurable in near real time may be useful, but these proposals require evaluation. One immediate innovation is a Trust Early-Warning System. Use a handful of live signals that are already collected such as same-team rebooking rate, missed/late result follow-up, repeat contacts within 72 h, and a one-question micro-survey (“Do you know who is responsible for your care right now?”). Here, “trust signals” refer to simple operational proxies (for example, follow-through and clarity of ownership) that may reflect perceived trustworthiness, rather than direct measures of trust. Combine them into a simple trust risk score for key cohorts. Before publishing the weekly rota, run a quick “what if” check: will the plan keep continuity above your minimum for those cohorts? If not, make small swaps (hold back some follow-up slots for the same team; avoid scattering one clinician across many sites) until the score improves. This is a light-touch way to plan the week around protecting trust, not just filling sessions.

Table 1 operationalises workforce instability by separating common patterns (turnover/short placements, rota volatility, understaffing, temporary staffing and cross-site fragmentation) and linking each to the continuity domain and trust mechanism most directly at risk. This approach is presented as a testable service-design hypothesis and can be assessed for feasibility, unintended consequences, and effects on patient experience and staff workload.

**Table 1:** Trust–continuity dashboard: operational responses to workforce instability.

Instability pattern	Trust mechanism at risk	Patient-visible signals	Design response (service-level)	Primary metrics (illustrative targets)	Data source & cadence	Accountable owner	First test-of-change (PDSA)
High turnover in a practice or service	Loss of relational continuity	“I never see the same person”; re-telling history	Empanelment and micro-teams; reserve follow-up capacity for team	UPC for complex cohort (aim ≥0.60) (16); GPPS confidence/trust stable vs. baseline (6, 7)	EHR panel lists monthly; GPPS annually	Clinical director; rostering lead	Allocate each patient to a named 3–4 person team; protect 20% team follow-up slots for 8 weeks
Rota gaps and cancelled clinics	Plan drift; weak follow-through	Late or changed appointments; missed result follow-up	Priority re-booking with same team ≤7 days; result “owner” list	7-day re-booking rate ≥85%; unattended result rate ≤1%	Scheduling & results logs weekly	Service manager	Auto-reschedule with “same-team” rule; daily result owner check
Heavy temporary staffing without induction	Fragmented informational continuity	Generic advice; inconsistent safety-netting	Standardised induction; access to care-plan templates; named team mentor	Handover completeness ≥95%; safety-net plan documented ≥90%	Monthly handover audit	Department lead; education lead	One-page induction + 10-case audit cycle
Short rotations with frequent handoffs	Accountability diffusion	“Who is responsible today?”	Named teams on ward; daily team brief; visible ownership boards	% patients naming their team ≥80%; escalation response within agreed timeframe	Bedside micro-surveys weekly; pager logs	Ward manager; consultant on-call	Introduce team boards on two bays; measure weekly for 6 weeks
Fragmented records across sites/apps	Loss of narrative memory	Contradictory advice; repeated history	Shared care plan and problem-list hygiene; “continuity note” capturing what matters	Care-plan completeness ≥85%; duplicate problem entries ≤5%	EHR reports monthly	IT clinical safety officer	Add standard care-plan template; pilot in two clinics
Remote-first models with weak introductions	Thin relational cues	Perceived indifference; uncertainty about next steps	Scripted team introduction; expectation setting; warm transfers to usual team	First-contact resolution ≥70%; GPPS trust stable vs. baseline (6, 7)	Call analytics monthly; GPPS annually	Access manager	Deploy call-opening script for one team; review 50 calls
Sickness-absence instability	Unplanned gaps and unfamiliar faces	Chaotic booking; repeated cancellations	Pre-defined intra-team cover matrix; flexible pool as last resort	% unplanned gaps covered within team ≥75%	Roster logs weekly	Rostering lead	Build cover matrix for three teams; monitor for two months
Out-of-hours to in-hours transition	Breaks in follow-through	“No one called me back”	Next-day team “hot list” with named reviewer; patient message with team ID	% OOH cases reviewed by owning team next day ≥90%	EHR tasking daily	Duty doctor lead	Create OOH-to-team hot-list protocol; track 30 consecutive cases
High-need cohorts (frailty, SMI, multimorbidity)	Disproportionate trust erosion	Avoidance, DNAs	priority assignment to continuity-focused teams; proactive contacts; longer slots	DNA rate ≤10%; 30-day crisis/readmission reduction	EHR + mental health dashboard monthly	Integrated care lead	Enrol first 50 patients into named teams; compare 3-month pre/post
Cross-cover on wards/clinics	Team mental model weak	Inconsistent plans; mixed messages	Daily huddles; standardised structured handover; fixed review times	% plans coherently updated across 48 h ≥ 90%	Handover review weekly	Clinical governance lead	Start 10 min huddles at set times; assess 20 cases

A further option is to introduce a 60-second “Continuity Note” at the end of each relevant consultation. This would involve recording a brief note or voice clip that captures the problem list, what matters to the patient, the plan agreed today and the safety-net should circumstances change. The Note remains visible to the next clinician and moves with the patient across settings. Coupling this with a simple Portable Team ID in all communications (e.g., “Your team: A2”) enables warm transfers to the appropriate team. Together, these tools help ensure that the patient's story and sense of ownership persist despite staff changes. Usage of the note and first-contact resolution should be monitored alongside continuity indices to assess impact.

A further option is to run weekly trust rounds at the same time each week (for example, a 15-minute huddle involving the duty clinician, a nurse or ACP representative, and a service manager or administrator), with one agreed action recorded as a small test of change (PDSA) and reviewed at the following meeting against the same two run-chart measures (priority-cohort continuity and the one-question trust response); where the team looks at three quick things: (1) one trust signal that worsened, (2) one patient story where handover worked well, and (3) one tiny change to test this week (e.g., auto-rebook with the same team within seven days after a cancellation). Publish a single run chart on the wall or intranet with two lines only: continuity for the priority cohort and the one-question trust response. Keep the bar low: no slides, no blame, just a visible rhythm of small changes, measured. Over a month, this creates a shared habit of designing for trust, not hoping for it.

## Discussion

Workforce instability is regularly described as a finance or throughput issue, though it has important relational consequences: it may undermine the environment in which patient trust is created and sustained. Instability in the near term is probably not going away, in spite of national growth and retention strategies and recurring regulatory scrutiny of persistent operational strain (7, 8, 11, 41, 43). The ethical response is not to blame clinicians for rota gaps, but to design for trust and access together.

Workforce stability is also a prerequisite for person-centred care for staff: stable teams make it easier to share responsibility, preserve narrative memory, and sustain the relational work of care without repeated 'starting again'. Conversely, persistent churn can increase cognitive load, supervision burden and moral distress, particularly when clinicians cannot provide the continuity they know patients need. Supporting retention and team stability therefore protects patients' experience of person-centred care while also improving workforce sustainability (1, 46, 47).

Trust develops when relationships are repeated, stories are remembered and plans remain coherent which are direct expressions of relational, informational and management continuity (20, 23, 24, 27, 39). Services should therefore anchor patients within small, named teams and normalise warm handovers that convey both narrative and intent, even when individual clinicians change. Measurement must make these practices visible: continuity indices such as the Usual Provider of Care quantify whether care is anchored; patient-reported confidence and trust items provide direct service-level signals; and policy analyses can help benchmark and target improvement where continuity has declined (12, 15, 22). Although causal inference is limited, the direction of associations is plausible and the consistency across settings strengthens the argument for continuity as a safety intervention (42, 43, 45).

The case for investment in promoting continuity of care extends well beyond its relational benefits. Observational studies consistently associate higher continuity with fewer ambulatory care–sensitive admissions, lower mortality, and reduced hospital use and costs (16, 18). Although causality cannot be established, the coherence of mechanisms and consistency of findings support treating continuity as a plausible safety strategy and one that may protect trust as well as outcomes. Such efforts should be prioritised for groups where the marginal benefit is greatest, including people with multimorbidity, frailty or severe mental illness, aligning continuity initiatives with the broader contribution of strong, person-centred primary care to population health and equity (25, 38, 40, 43). Retention is also a trust strategy: predictable clinician patterns, adequate supervision and supportive cultures help staff thrive and reduce the relationship ruptures that patients experience (8, 37, 39).

### Future directions

The next step is to embed a light digital trust infrastructure into routine systems: a visible “trust thread” within the record that documents who last made which commitments, when the next follow-up is due, and a single-question prompt such as “Do you know who is responsible for your care right now?” captured at key touchpoints. This would make trust auditable in real time rather than inferred only after problems occur, and it would align operational decisions such as rostering and handovers with a live trust signal.

Regulators and commissioners could consider treating continuity and trust as reportable standards, published alongside access and safety, where measurement burden is proportionate and data quality is sufficient. A paired run chart plotting continuity for priority cohorts and responses to the one-question trust prompt would reveal where instability erodes confidence despite good headline access. Public reporting would help re-centre improvement efforts on the relational foundations of safe care, not solely on throughput.

Finally, we may adopt scalable, low-effort practices that maintain ownership despite changing personnel. Examples include a 60-second Continuity Note capturing key elements of the consultation, a team identifier on all communications to enable warm transfers, and brief weekly “trust rounds” to review signals, successes and small tests of change. These strategies are adaptable across systems, even those with persistent turnover. The goal is not to eliminate instability but to embed trust into routine processes so that continuity and accountability remain clear to patients.

Workforce turbulence may continue; loss of trust and loss of person-centred care are not inevitable. Stable teams, reliable handovers and transparent continuity and trust metrics can protect patients from the effects of instability and uphold the relational basis of safe, equitable care.
